# Neural Representation of Concurrent Vowels in Macaque Primary Auditory Cortex^1^,^2^,^3^

**DOI:** 10.1523/ENEURO.0071-16.2016

**Published:** 2016-06-10

**Authors:** Yonatan I. Fishman, Christophe Micheyl, Mitchell Steinschneider

**Affiliations:** 1 Departments of Neurology and Neuroscience, Albert Einstein College of Medicine, Bronx, New York 10461; 2 Department of Psychology, University of Minnesota, Minneapolis, Minnesota 55455; 3 Starkey Hearing Research Center, Berkeley, California 94704

**Keywords:** auditory scene analysis, multiunit activity, pitch, speech perception

## Abstract

Successful speech perception in real-world environments requires that the auditory system segregate competing voices that overlap in frequency and time into separate streams. Vowels are major constituents of speech and are comprised of frequencies (harmonics) that are integer multiples of a common fundamental frequency (F0). The pitch and identity of a vowel are determined by its F0 and spectral envelope (formant structure), respectively. When two spectrally overlapping vowels differing in F0 are presented concurrently, they can be readily perceived as two separate “auditory objects” with pitches at their respective F0s. A difference in pitch between two simultaneous vowels provides a powerful cue for their segregation, which in turn, facilitates their individual identification. The neural mechanisms underlying the segregation of concurrent vowels based on pitch differences are poorly understood. Here, we examine neural population responses in macaque primary auditory cortex (A1) to single and double concurrent vowels (/a/ and /i/) that differ in F0 such that they are heard as two separate auditory objects with distinct pitches. We find that neural population responses in A1 can resolve, via a rate-place code, lower harmonics of both single and double concurrent vowels. Furthermore, we show that the formant structures, and hence the identities, of single vowels can be reliably recovered from the neural representation of double concurrent vowels. We conclude that A1 contains sufficient spectral information to enable concurrent vowel segregation and identification by downstream cortical areas.

## Significance Statement

The ability to attend to a particular voice among competing voices is crucial for speech perception in complex acoustic environments. This ability requires that listeners perceptually segregate sounds into discrete auditory streams, which correspond to their sources. Vowels are major constituents of speech. The pitch and identity of a vowel are determined by its fundamental frequency and spectral envelope (formant structure), respectively. A difference in pitch between two simultaneous vowels provides a powerful cue for their segregation, which in turn, facilitates their individual identification. Here, we show that primary auditory cortex contains sufficient spectral information to enable concurrent vowel segregation and identification based on differences in pitch by downstream cortical areas, consistent with its role in auditory scene analysis.

## Introduction

The ability to selectively attend to a particular voice among competing voices is crucial for speech perception in complex real-world acoustic environments (e.g., a noisy restaurant). This ability requires that listeners first analyze the auditory scene and perceptually segregate sounds into discrete auditory streams corresponding to each of the talkers, which can then be selected for further processing by top-down attentional mechanisms (Shinn-Cunningham and Best, 2008; Kong et al., 2015). Sound segregation is a computationally challenging task, given that sounds in real-world environments typically overlap in both frequency and time.

The neural mechanisms underlying concurrent sound segregation remain unclear. Several lines of evidence support a role for auditory cortex in concurrent sound segregation (Alain et al., 2005; Alain, 2007; Bidet-Caulet et al., 2007, 2008) and in selective attention to particular streams of concurrent speech (Ding and Simon, 2012; Mesgarani and Chang, 2012; Zion-Golumbic et al., 2013; Kong et al., 2015). However, the ability to attend to one speech stream in the midst of other competing streams critically depends on how well they are perceptually segregated from one another (Shinn-Cunningham and Best, 2008; Kong et al., 2015). The key question of how the auditory system segregates concurrent, spectrally overlapping, speech streams prior to their attentional selection remains unanswered.

A difference in pitch between two simultaneous voices provides a powerful cue for their segregation. Vowels are major constituents of speech and are comprised of frequencies (harmonics) that are integer multiples of a common fundamental frequency (F0), which defines their pitch. When two spectrally overlapping vowels differing in F0 are presented concurrently, they can be readily perceived as two separate “auditory objects” with distinct pitches at their respective F0s. Identification of concurrent vowels and their pitches is facilitated by an increase in the difference in F0 between them (Assmann and Summerfield, 1990; Chalikia and Bregman, 1993; Culling and Darwin, 1994; de Cheveigné et al., 1995; Assmann and Paschall, 1998).

Importantly, the perceptual segregation of concurrent vowels is greatly enhanced when their harmonics are individually resolved by the auditory system (Oxenham, 2008; Micheyl and Oxenham, 2010). We have previously shown that neuronal populations in monkey primary auditory cortex (A1) can resolve the lower harmonics of single and double concurrent harmonic complex tones (HCTs) via a “rate-place” code, a prerequisite for deriving their pitches and for their perceptual segregation based on spectral cues (Fishman et al., 2013, 2014). In principle, the pitches of the HCTs can be extracted from these rate-place representations via “harmonic templates”, perhaps implemented by “pitch-selective” neurons in non-primary auditory cortex that receive input from A1 (Bendor and Wang, 2005). Unlike HCTs with flat spectral envelopes, vowels contain peaks in their spectral envelopes, ie, formants, which determine the identity of the vowels and contribute to their discrimination. Several studies have shown that rate-place codes in A1 can reliably represent the formant structure of single, isolated vowels (Mesgarani et al., 2008; Qin et al., 2008; Walker et al., 2011). However, it remains unknown whether A1 can represent both the harmonics (spectral fine-structure) and the formants (spectral envelopes) of concurrent vowels differing in F0 with sufficient resolution to enable their perceptual segregation and identification.

Here, we examine the neural representation of single and double concurrent vowels in A1 of alert macaque monkeys. F0s of double vowels differed by four semitones, an amount sufficient for them to be heard as two separate auditory objects with distinct pitches by human listeners (Assmann and Summerfield, 1990; Assmann and Paschall, 1998). We find that neural population responses can resolve lower harmonics of both single and double concurrent vowels. Furthermore, we show that the spectral envelopes (formant structures), and hence identities, of single vowels can be reliably recovered from the neural representation of double concurrent vowels. We conclude that A1 contains sufficient spectral information to enable concurrent vowel segregation and identification based on differences in pitch by downstream cortical areas receiving the output of A1.

## Materials and Methods

Neurophysiological data were obtained from three adult male macaque monkeys (*Macaca fascicularis*) using previously described methods (Steinschneider et al., 2003; Fishman and Steinschneider, 2010). All experimental procedures were reviewed and approved by the AAALAC-accredited Animal Institute of Albert Einstein College of Medicine and were conducted in accordance with institutional and federal guidelines governing the experimental use of nonhuman primates. Animals were housed in our AAALAC-accredited Animal Institute under daily supervision of laboratory and veterinary staff. Prior to surgery, monkeys were acclimated to the recording environment and were trained to perform a simple auditory discrimination task (see below) while sitting in custom-fitted primate chairs.

### Surgical procedure

Under pentobarbital anesthesia and using aseptic techniques, rectangular holes were drilled bilaterally into the dorsal skull to accommodate epidurally placed matrices composed of 18 gauge stainless steel tubes glued together in parallel. Tubes served to guide electrodes toward auditory cortex for repeated intracortical recordings. Matrices were stereotaxically positioned to target A1 and were oriented to direct electrode penetrations perpendicular to the superior surface of the superior temporal gyrus, thereby satisfying one of the major technical requirements of one-dimensional current source density (CSD) analysis (Müller-Preuss and Mitzdorf, 1984; Steinschneider et al., 1992). Matrices and Plexiglas bars, used for painless head fixation during the recordings, were embedded in a pedestal of dental acrylic secured to the skull with inverted bone screws. Perioperative and postoperative antibiotic and anti-inflammatory medications were always administered. Recordings began after at least 2 weeks of postoperative recovery.

### Stimuli

Stimuli were generated and delivered at a sample rate of 48.8 kHz by a PC-based system using an RX8 module (Tucker Davis Technologies). Frequency response functions derived from responses to pure tones characterized the spectral tuning of the cortical sites. Pure tones used to generate the frequency response functions ranged from 0.15 to 18.0 kHz, were 200 ms in duration (including 10 ms linear rise/fall ramps), and were randomly presented at 60 dB SPL with a stimulus onset-to-onset interval of 658 ms. Resolution of frequency response functions was 0.25 octaves or finer across the 0.15 to 18.0 kHz frequency range tested.

All stimuli were presented via a free-field speaker (Microsatellite, Gallo) located 60° off the midline in the field contralateral to the recorded hemisphere and 1 m away from the animal’s head (Crist Instruments). Sound intensity was measured with a sound level meter (type 2236; Bruel and Kjaer) positioned at the location of the animal’s ear. The frequency response of the speaker was flat (within ±5 dB SPL) over the frequency range tested.

### General approach

**Figure 1.**
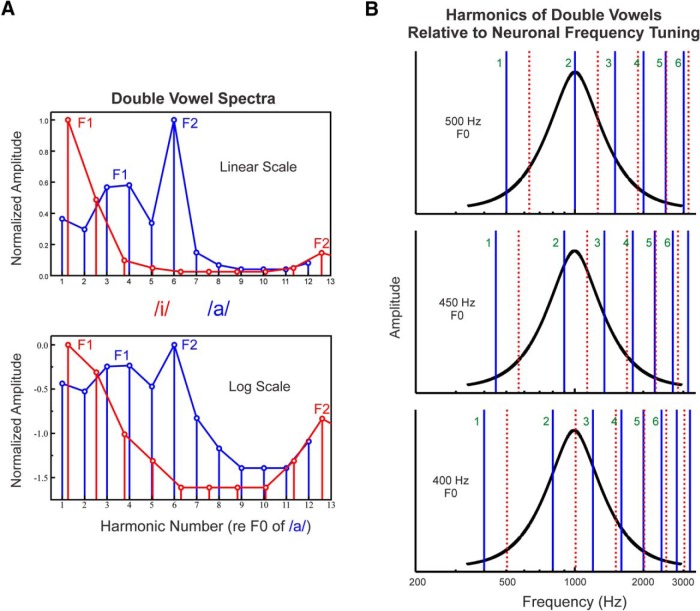
Schematic representation of the double vowel stimuli presented in the study. ***A***, Spectra of double vowel stimuli plotted on both linear and logarithmic scales. Stimulus amplitude and frequency are represented along the vertical and horizontal axes, respectively. Stimuli consisted of a series of two simultaneously presented vowels, /a/ and /i/, with a fixed F0 difference between them of four semitones (a major 3rd). Harmonics of the vowel with the lower F0 (/a/) and higher F0 (/i/) are represented by the vertical blue and red drop lines, respectively. The spectral envelopes of the vowels are represented by the lines connecting the vertical drop lines. Main formants of the vowels (peaks in the spectral envelopes) are labeled. ***B***, Harmonics of double vowels relative to neuronal frequency tuning. Harmonics of the vowel with the lower F0 (/a/) and higher F0 (/i/) are represented by the solid blue and broken red lines, respectively. All harmonics are shown at equal amplitude for clarity. The F0 of the vowel with the lower pitch is varied such that harmonics of the double vowel fall progressively on either the peak (at the BF, here equal to 1000 Hz) or the sides of the neuronal frequency response function (black). As the F0 of the higher-pitched vowel (/i/) is fixed at four semitones above the F0 of the lower-pitched vowel (/a/), the F0 of the higher-pitched vowel varies correspondingly. The F0 of the vowel /a/ is indicated on the left of each plot; the first six harmonics of /a/ are labeled. If individual harmonics of the double vowel stimuli can be resolved by frequency-selective neurons in A1, then response amplitude as a function of F0 (or harmonic number: BF/F0) should display peaks when a given harmonic of /a/ or /i/ overlaps the BF (top and bottom plots) and troughs when the BF falls in between two adjacent harmonics of the concurrent vowels (middle plot).

We examined neural representations of single and double concurrent vowels (/a/ and /i/, as in “father” and “street”, respectively) in A1 of alert monkeys via a stimulus design used in studies of HCT encoding in the auditory nerve (Larsen et al., 2008) and cortex (Fishman et al., 2013, 2014). Each vowel was comprised of 12 harmonics (maximum level of each harmonic 60 dB SPL; 225 ms total stimulus duration). Double vowels were created by summation of the two single vowels. Spectral envelopes (formants) of the vowels with respect to harmonic number were shaped using a Klatt synthesizer (Fig. 1). F0s of the double vowels differed by a ratio of 1.26 or 4 semitones (with /i/ at the higher F0), an amount sufficient for them to be perceived by human listeners as two separate auditory objects with distinct pitches at their respective F0s and reliably identified by their distinct formant structure (Assmann and Summerfield, 1990; Culling and Darwin, 1993; de Cheveigné, 1999; Assmann and Paschall, 1998; Snyder and Alain, 2005).

F0s of the vowels varied in increments of 1/8 harmonic number relative to the best frequency (BF; defined below) of the recorded neural populations to generate rate-place representations of the single and double vowels (where “harmonic number” is defined as BF/F0). By systematically varying the F0, harmonics progressively fall either on the peak or on the sides of the neuronal pure-tone tuning functions (Fig. 1). The resulting neural response amplitude versus harmonic number functions are called rate-place profiles (terminology from Cedolin and Delgutte, 2005; Larsen et al., 2008; see Discussion for relevant caveats).

A set of 89 vowel stimuli with variable F0s was used to generate each rate-place profile. The vowel /a/ with the highest F0 was configured such that its F0 matched the BF of the site, whereas that with the lowest F0 was configured such that its highest (12th) harmonic matched the BF. Sampling at increments of 1/8 harmonic number in the current study has yielded rate-place profiles with sufficient resolution to resolve lower harmonics of HCTs with flat-spectral envelopes both in auditory nerve and cortical recordings (Cedolin and Delgutte, 2005; Larsen et al., 2008; Fishman et al., 2013, 2014).

The above-described stimulus protocol typically required 3–4 h of recording time to complete. This timeframe precluded testing more than one F0 separation or pair of vowels in a given electrode penetration, which would have required prohibitively long neurophysiological recording sessions in behaving monkeys. The use of a four-semitone F0 separation allowed us to determine whether A1 can resolve harmonics and spectral envelopes of single and double vowels at the minimum F0 separation required for human subjects to reliably hear the concurrent vowels as two separate sounds with distinct pitches and to identify them (Assmann and Paschall, 1998).

### Neurophysiological recordings

Recordings were conducted in an electrically shielded, sound-attenuated chamber. Monkeys were monitored via video camera throughout each recording session. To promote attention to the sounds during the recordings, animals performed a simple auditory discrimination task (detection of a randomly presented noise burst interspersed among test stimuli) to obtain liquid rewards. An investigator entered the recording chamber and delivered preferred treats to the animals prior to the beginning of each stimulus block to further maintain alertness of the subjects.

Local field potentials (LFPs) and multiunit activity (MUA) were recorded using linear-array multi-contact electrodes comprised of 16 contacts, evenly spaced at 150 μm intervals (U-Probe, Plexon). Individual contacts were maintained at an impedance of ∼200 kΩ. An epidural stainless-steel screw placed over the occipital cortex served as the reference electrode. Neural signals were band-pass filtered from 3 Hz to 3 kHz (roll-off 48 dB/octave), and digitized at 12.2 kHz using an RA16 PA Medusa 16-channel preamplifier connected via fiber-optic cables to an RX5 data acquisition system (Tucker-Davis Technologies). LFPs time-locked to the onset of the sounds were averaged on-line by computer to yield auditory evoked potentials (AEPs). CSD analyses of the AEPs characterized the laminar distribution of net current sources and sinks within A1, and were used to identify the laminar location of concurrently recorded AEPs and MUA (Steinschneider et al., 1992, 1994). CSD was calculated using a three-point algorithm that approximates the second spatial derivative of voltage recorded at each recording contact (Freeman and Nicholson, 1975; Nicholson and Freeman, 1975).

MUA was derived from the spiking activity of neural ensembles recorded within lower lamina 3, as identified by the presence of a large amplitude initial current sink that is balanced by concurrent superficial sources in mid-upper lamina 3 (Steinschneider et al., 1992; Fishman et al., 2001). This current dipole configuration is consistent with the synchronous activation of pyramidal neurons with cell bodies and basal dendrites in lower lamina 3. Previous studies have localized the initial sink to the thalamorecipient zone layers of A1 (Müller-Preuss and Mitzdorf, 1984; Steinschneider et al., 1992; Sukov and Barth, 1998; Metherate and Cruikshank, 1999). To derive MUA, filtered neural signals (3 Hz to 3 kHz pass-band) were subsequently high-pass filtered at 500 Hz (roll-off 48 dB/octave), full-wave rectified, and then low-pass filtered at 520 Hz (roll-off 48 dB/octave) prior to averaging of single-trial responses (for review, see Supèr and Roelfsema, 2005). MUA is a measure of the envelope of summed (synchronized) action potential activity of local neuronal ensembles (Vaughan and Arezzo, 1988; Brosch et al., 1997; Schroeder et al., 1998; Supèr and Roelfsema, 2005; O’Connell et al., 2011). Thus, while firing rate measures are typically based on threshold crossings of neural spikes, MUA, as derived here, is an analog measure of spiking activity in units of response amplitude (Kayser et al., 2007). MUA and single-unit activity, recorded using electrodes with an impedance similar to that in the present study, display similar orientation and frequency tuning in primary visual and auditory cortex, respectively (Supèr and Roelfsema, 2005; Kayser et al., 2007). Adjacent neurons in A1 (ie, within the sphere of recording for MUA) typically display synchronized responses with similar spectral tuning; a fundamental feature of local processing that may promote high-fidelity transmission of stimulus information to subsequent cortical areas (Atencio and Schreiner, 2013). Thus, MUA measures are appropriate for examining the neural representation of spectral cues in A1, which may be used by neurons in downstream cortical areas for pitch extraction and concurrent sound segregation (Bendor and Wang, 2005).

Positioning of electrodes was guided by on-line examination of click-evoked AEPs and the derived CSD profile. Pure tone stimuli were delivered when the electrode channels bracketed the inversion of early AEP components and when the largest MUA and initial CSD current sink were situated in middle channels. Evoked responses to about 40 presentations of each pure tone or vowel stimulus were averaged with an analysis time of 500 ms that included a 100 ms prestimulus baseline interval. The BF of each cortical site was defined as the pure tone frequency eliciting the maximal MUA within a time window of 0–75 ms poststimulus onset. This response time window includes the transient “On” response elicited by sound onset and the decay to a plateau of sustained activity in A1 (Fishman and Steinschneider, 2009). Following determination of the BF, vowel stimuli were presented.

At the end of the recording period, monkeys were deeply anesthetized with sodium pentobarbital and transcardially perfused with 10% buffered formalin. Tissue was sectioned in the coronal plane (80 μm thickness) and stained for Nissl substance to reconstruct the electrode tracks and to identify A1 according to previously published physiological and histological criteria (Merzenich and Brugge, 1973; Morel et al., 1993; Kaas and Hackett, 1998). Based upon these criteria, all electrode penetrations considered in this report were localized to A1, though the possibility that some sites situated near the low-frequency border of A1 were located in field R cannot be excluded.

### Analysis and interpretation of responses to single and double vowels

Responses to double vowels were analyzed with the aim of determining whether A1 contains sufficient spectral information to reliably extract the F0s of each of the vowels based on their harmonic fine structure and to identify them based on their formant structure. The former requires sufficient neuronal resolution of individual harmonics of the vowels (Plack et al., 2005), whereas the latter requires accurate neural representation of their spectral envelopes.

**Figure 2.**
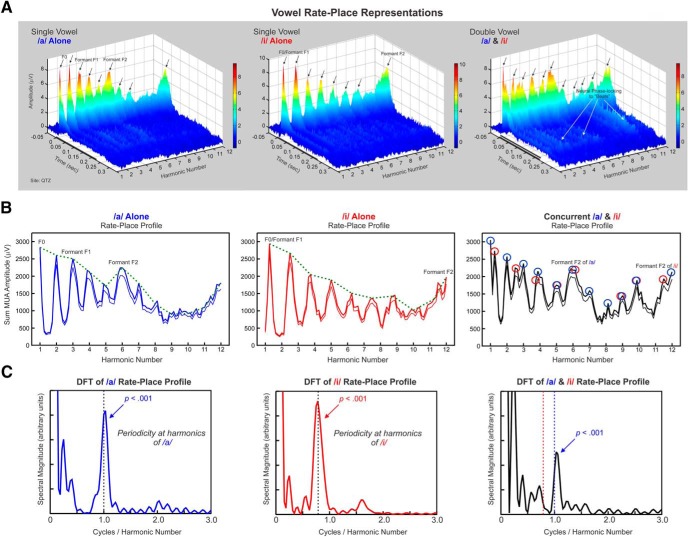
Example rate-place representations of single and double concurrent vowels. ***A***, Rate-place representations of single vowels (left and middle plots, /a/ and /i/, respectively) and double vowels (right plot) based on neuronal responses recorded at a site with a BF of 5750 Hz. Axes represent harmonic number (BF/F0 of the vowel /a/), time, and response amplitude in microvolts (also color-coded), as indicated. The black bars represent the duration of the stimuli (225 ms). In rate-place representations of single vowels, amplitude of On and Sustained activity displays a periodicity with prominent peaks (indicated by black arrows) occurring at or near values of harmonic number corresponding to the frequency components of the stimuli. Peaks corresponding to vowel formants are indicated. In rate-place representations of double vowels, peaks in the amplitude of On responses (indicated by black arrows) occur at or near values of harmonic number corresponding to frequency components of each of the vowels. Neuronal phase-locking to “beats” (stimulus waveform amplitude fluctuations indicated by white arrows) is also evident in the rate-place representation of the double vowels. ***B***, Corresponding rate-place profiles of single and double vowels (as indicated) based on the area under the MUA waveform within the On time window. The thick lines represent the mean MUA, whereas the thin lines represent 1 SE below the mean. Envelopes of rate-place profiles are represented by the green dashed lines. Peaks in neural activity occur at or near values of harmonic number corresponding to the frequency components of the vowels. Peaks in the rate-place profile of the double vowel occurring at or near frequency components of /a/ and /i/ are indicated by the blue and red circles, respectively. ***C***, Corresponding DFTs of the rate-place profiles shown in ***B***.

At each recording site, the F0 of the lower-pitched vowel (/a/) of the double-vowel stimuli was varied in small increments, such that harmonics fell either on the peak, or in a progressive manner, on the sides of the neuronal pure-tone tuning functions (Fig. 1). As the F0 of the higher-pitched vowel (/i/) of the double-vowel stimuli was always fixed at four semitones (a major 3rd) above the F0 of the lower-pitched vowel, the F0 of the higher-pitched vowel varied correspondingly. If harmonics of the lower-pitched vowel can be resolved by patterns of neuronal firing across frequency-selective neurons in A1, then rate-place profiles should display a periodicity with peaks occurring at integer multiples of 1.0. Similarly, if A1 neurons can resolve harmonics of the vowel with the higher F0, then rate-place profiles should display a periodicity with peaks occurring at integer multiples of 1.26 (corresponding to a ratio of F0s differing by 4 semitones). Neural representation of the harmonic spectra of the single and double vowels, as reflected by periodicity in the rate-place profile, was evaluated by computing the discrete Fourier transform (DFT) of the rate-place profile. The salience of the periodicity is quantified by the amplitude of the peak in the DFT at a frequency of 1.0 cycle/harmonic number (for harmonics of /a/) and of 1/1.26=0.79 cycle/harmonic number (for harmonics of /i/). Statistical significance of peaks in the DFTs was assessed using a nonparametric permutation test (Ptitsyn et al., 2006; Womelsdorf et al., 2007; Fishman et al., 2013, 2014), which entails randomly shuffling the data points in the rate-place profile, computing the DFT of the shuffled data, and measuring the spectral magnitude at 1.0 cycle/harmonic number and at 0.79 cycle/harmonic number. This method of quantification is illustrated in Figure 2*C*. Repeating this process 1000 times yields a “null” distribution of spectral magnitudes. The probability of obtaining a spectral magnitude equal to or greater than the value observed, given the null, can be estimated by computing the proportion of the total area under the distribution at values equal to and greater than the observed value (Fishman et al., 2013, 2014). Spectral magnitude values yielding *p* values below 0.05 were considered statistically significant and interpreted as indicating neural representation of the vowel harmonics via a rate-place code.

Two response time windows were analyzed: On (0–75 ms) and “Sustained” (75–225 ms). Our previous studies of responses to single and double HCTs with flat spectral envelopes showed that On responses resolved individual harmonics of the HCTs better than Sustained responses (Fishman et al., 2013, 2014). As a similar trend was observed for responses to vowel stimuli, the present report focuses exclusively on the On portion of responses.

To determine whether rate-place profiles in A1 reliably reflect the spectral envelopes (formant structures) of the individual vowels, we computed the Pearson correlation between the envelope of the rate-place profiles and the spectral envelopes of each of the two vowel stimuli. The envelope of the rate-place profile for each vowel was obtained by measuring the amplitude in the rate-place profiles at values of harmonic number corresponding to each of the two vowels (Fig. 2*B*, green dashed lines). Good vowel identification is reflected by a high correlation between the envelope of the rate-place profile elicited by a given vowel and the spectral envelope of the corresponding (matching) vowel stimulus. On the other hand, good vowel discrimination is reflected by a low correlation between the envelope of the rate-place profile elicited by a given vowel and the spectral envelope of the other (non-matching) vowel stimulus.

## Results

Results are based on MUA recorded in 18 multicontact electrode penetrations into A1 of three monkeys. Interim analyses of these 18 recording sites yielded statistically reliable results (alpha level =0.05), which were deemed sufficient to achieve the objectives of the study. Limiting the number of electrode penetrations thereby maximized the use of the monkeys for additional experiments. MUA data presented in this report were recorded from a single electrode contact positioned within lower lamina 3, the layer typically displaying the largest neural responses in A1 and likely reflecting pyramidal neuron activity (Steinschneider et al., 1992). Four additional sites were excluded from analysis because they did not respond to any of the vowel stimuli presented, were characterized by “Off”-dominant responses, had aberrant CSD profiles that precluded adequate assessment of laminar structure, or displayed frequency response functions that were too broad to accurately determine a BF (a prerequisite for determining the vowel F0s used to generate rate-place profiles). Sites that showed broad frequency tuning were situated along the lateral border of A1.

For all sites examined, responses occurring within the On response time window (0–75 ms poststimulus onset) displayed sharp frequency tuning characteristic of small neural populations in A1 (Fishman and Steinschneider, 2009). Mean MUA onset latency and mean 6 dB bandwidths of MUA frequency response functions were ∼14 ms and ∼0.6 octaves, respectively. These values are comparable to those reported for single neurons in A1 of awake monkeys (Recanzone et al., 2000). Although this onset latency is more consistent with activation of cortical neuron populations than with thalamocortical fiber activity (Steinschneider et al., 1992; 1998; our unpublished observations), the possibility that spikes from thalamocortical afferents contributed to our response measures cannot be excluded. BFs of recording sites ranged from 250 to 16,000 Hz.

### Neural population responses in A1 can resolve the lower harmonics of single and double concurrent vowels

Figure 2*A* shows rate-place representations of single and double vowels based on MUA recorded at a representative site. Each rate-place representation is a composite of individual averaged responses to 89 single or double vowels depicted as a three-dimensional plot, with axes representing harmonic number (BF/F0) with respect to the F0 of /a/, time, and response amplitude (also color coded). Periodicity with respect to harmonic number is evident in the rate-place representations of single vowels, such that peaks in response amplitude (indicated by the black arrows) occur at values of harmonic number at which a spectral component of a given vowel, /a/ or /i/, matches the BF of the site. The rate-place representation of double vowels also shows prominent peaks in response amplitude occurring at harmonic number values corresponding to the spectral components of each of the vowels.

Associated rate-place profiles based on the area under the MUA waveform within the On time window are shown in Figure 2*B*. Periodic peaks in rate-place profiles corresponding to harmonics of /a/ and /i/ are enclosed by blue and red circles, respectively, when the vowels are presented simultaneously. Consistent with previous findings relating to the neural representation of single and double HCTs with flat spectral envelopes (Fishman et al., 2013, 2014), periodicity in the rate-place profiles for responses to double vowels is more prominent at lower harmonic numbers (1–6) than at higher harmonic numbers (7–12), thus indicating a greater capacity of neural responses to resolve the lower harmonics of the vowels. This observation is consistent with human psychoacoustic data indicating that lower harmonics are more important for pitch perception than higher harmonics (Micheyl and Oxenham, 2010).

Periodicity at 1.0 cycle/harmonic number in the rate-place profiles based on responses elicited by single vowels is statistically significant, as evaluated via a nonparametric permutation test based on the DFT of the rate-place profile (Fig. 2*C*, left; *p* < 0.001; see Materials and Methods for details). Significant periodicity at or near 1.0 cycle/harmonic number (permutation test; *p* < 0.001) is also observed in the rate-place profile for responses to double vowels (Fig. 2*C*, right), thus demonstrating the ability of this site to resolve the lower harmonics of /a/ when presented concurrently with /i/. However, at this site, periodicity corresponding to harmonics of /i/was not statistically significant when /a/ and /i/ were presented concurrently. As human subjects can reliably identify the pitches of both vowels when they are presented concurrently at a four-semitone F0 separation (Assmann and Paschall, 1998), it is important to consider neural responses across multiple sites in A1, which are likely to be more relevant for perception than responses at a single site (Bizley et al., 2010).

**Figure 3.**
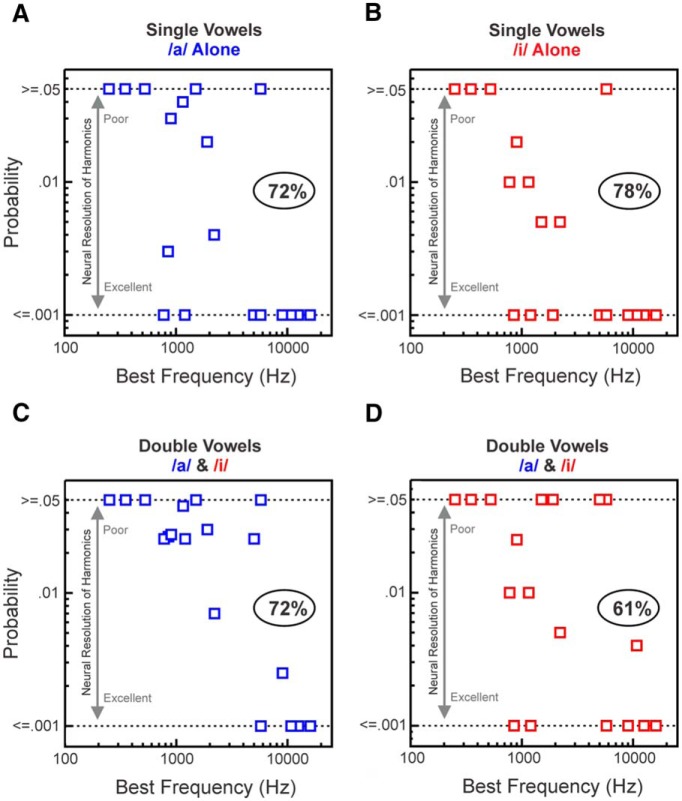
Neural population responses in A1 can represent the individual harmonics (spectral fine-structure) of single and double vowels. Periodicity in rate-place profiles of responses to single and double vowels, which reflects the neural representation of harmonics, is quantified by the amplitude of peaks in the DFT of rate-place profiles (Fig. 2). Statistical significance of peaks is evaluated via permutation tests. Estimated probabilities of the observed periodicity in rate-place profiles of responses to single and double vowels, given the null distribution derived from random shuffling of points in rate-place profiles, are plotted as a function of BF. Results for single vowels are shown in ***A*** and ***B*** (harmonics of /a/ and /i/, respectively) and results for double vowels are shown in ***C*** and ***D*** (harmonics of /a/ and /i/, respectively). Only results based on rate-place data corresponding to harmonic numbers 1–6 are shown (see text for explanation). Lower probability values indicate greater periodicity at 1.0 cycle/harmonic number (corresponding to harmonics of /a/) and at 0.79 cycle/harmonic number (corresponding to harmonics of /i/), and a correspondingly greater capacity of neural responses to resolve individual harmonics of the vowels. As probability values >0.05 are considered nonsignificant, for display purposes, values ≥0.05 are plotted along the same row, as marked by the upper horizontal dashed line at 0.05 along the ordinate. As permutation tests were based on 1000 shuffles of rate-place data, probability values <0.001 could not be evaluated. Therefore, probability values ≤0.001 are plotted along the same row, as marked by the lower horizontal dashed line at 0.001 along the ordinate. Numbers in ovals indicate the percentage of sites displaying statistically significant (*p* < 0.05) periodicity in rate-place profiles corresponding to harmonics of the vowels.

To evaluate the capacity of A1 to represent the harmonics of single and double vowels across all sites sampled, the estimated probability of the observed periodicity in rate-place profiles is plotted as a function of BF in Figure 3. Results corresponding to the rate-place representation of harmonics of single vowels are shown in Figure 3, *A* and *B*, (/a/ and /i/, respectively) and to the rate-place representation of harmonics of each of the two vowels when presented concurrently are shown in *C* and *D*. As results for double vowel harmonics 7–12 were not statistically significant, only those for harmonics 1–6 are shown. Lower probability values indicate greater periodicity in rate-place profiles at harmonics of /a/ and /i/ (1.0 and 0.79 cycle/harmonic number, respectively) and a correspondingly greater capacity of neural responses to resolve individual harmonics of the vowels. Numbers in the ovals indicate the percentage of sites displaying statistically significant (*p* < 0.05) periodicity in rate-place profiles. Although harmonics of single vowels were better resolved than those of double vowels, neural populations in A1 were still able to reliably resolve lower harmonics of each of the vowels comprising the double-vowel stimuli. Consistent with findings in the cat auditory nerve (Larsen et al., 2008) and in macaque A1 based on responses to HCTs with flat spectral envelopes (Fishman et al., 2013, 2014), sites with higher BFs generally resolved lower harmonics (1–6) of both single and double vowels better than sites with lower BFs. This is not surprising given that higher BF sites tend to display narrower relative tuning bandwidths than lower BF sites (ratio of frequency response function bandwidth to BF; Fishman and Steinschneider, 2009). Nonetheless, harmonics of at least one of the vowels in the double-vowel stimuli could still be clearly resolved at sites with BFs as low as 775 Hz.

### Neural population responses in A1 can identify and discriminate single and double concurrent vowels based on their spectral envelopes (formant structure)

**Figure 4.**
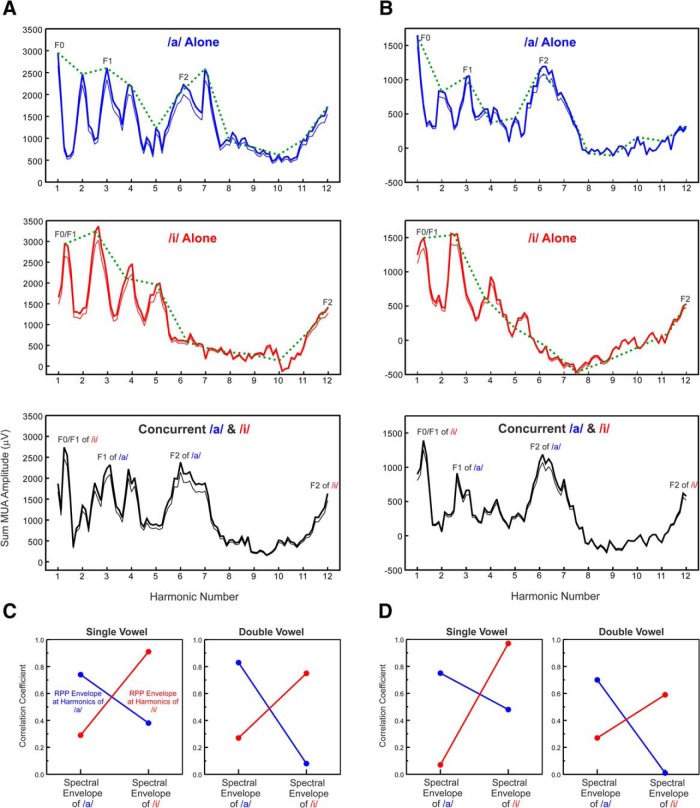
Representative rate-place profiles of responses to single and double concurrent vowels. Rate-place profiles of responses to single and double vowels recorded at two sites with BFs of 1200 and 850 Hz (***A*** and ***B***, respectively). Same conventions as in Figure 2. Major peaks corresponding to the first and second formants of the vowels are labeled. Pearson correlation between envelopes of the rate-place profiles (RPPs) at harmonics of the vowels and the corresponding spectral envelopes of the single vowel stimuli (Fig. 1) are shown in ***C*** and ***D*** for each of the two sites, respectively. For both single and double vowels, rate-place profile envelopes at harmonics of each of the vowels (/a/, blue lines; /i/, red lines) are highly correlated with the spectral envelopes of the matching vowel stimuli, whereas they are poorly correlated with the spectral envelopes of the non-matching vowel stimuli, thereby indicating that A1 responses can be used to identify and discriminate the vowels, both when presented in isolation and concurrently.

**Figure 5.**
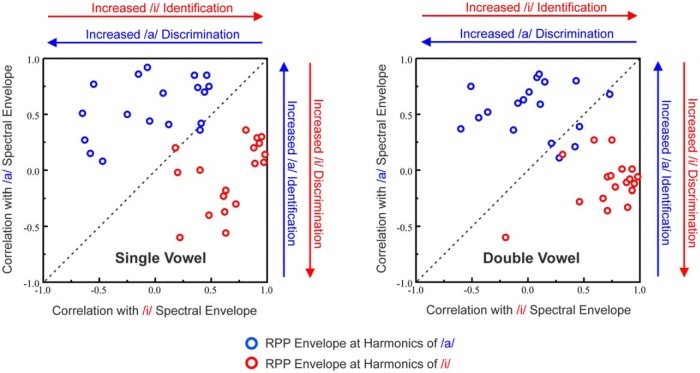
Neural population responses in A1 can identify and discriminate vowels based on their spectral envelopes (formant structure), both when presented alone and concurrently. Plot of Pearson coefficients of correlation between envelopes of rate-place profiles (RPPs) elicited by single and double vowels and spectral envelopes of the vowel stimuli /a/ and /i/ (left plot, single vowels; right plot, double vowels). Values for responses to /a/ and /i/ are plotted in blue and red, respectively. Good vowel identification is reflected by the high correlation between the envelope of the rate-place profile for a given vowel and the spectral envelope of the matching vowel stimulus. Good vowel discrimination is reflected by the low correlation between the envelope of the rate-place profile for a given vowel and the spectral envelope of the non-matching vowel stimulus.

Figure 4 shows rate-place profiles elicited by single and double vowels at two additional representative recording sites (*A* and *B*, respectively). As illustrated earlier, rate-place profiles elicited by single vowels can resolve the lower harmonics of the vowels. In addition, the rate-place profiles for single vowels display peaks at or near harmonic number values corresponding to the formants of the vowels (Fig. 4). These peaks persist in rate-place profiles elicited by the concurrent vowels. To examine whether rate-place profiles elicited by the single and concurrent double vowels can identify and discriminate between the vowels, we computed the Pearson correlation between the envelopes of the rate-place profiles at harmonics of the vowels (green dashed lines) and the spectral envelopes of the single vowel stimuli (Fig. 1). Note that points connected by the green dashed lines represent the amplitude in the rate-place profiles at values of harmonic number corresponding to each of the two vowels. Thus, peaks in the green curves need not match, and indeed may underestimate, the amplitude of peaks in the rate-place profiles. For both sites shown, rate-place profile envelopes at harmonics of each of the vowels were highly correlated (*p* ≤ 0.05) with the spectral envelopes of the matching vowels (eg, rate-place envelope of /a/ compared with the stimulus envelope of /a/; Fig. 4*C*,*D*). On the other hand, rate-place profile envelopes were comparatively poorly correlated with the spectral envelopes of the non-matching vowels (eg, rate-place envelope of /a/ compared with the stimulus envelope of /i/). This pattern of high and low correlations indicates that rate-place representations in A1 can be used to identify and discriminate, respectively, both single and concurrent vowels based on their formant structures. A similar pattern of high and low correlations, reflecting reliable vowel identification and discrimination based on A1 responses to both single and concurrent vowels, is observed across the entire sample of recording sites (Fig. 5).

**Figure 6.**
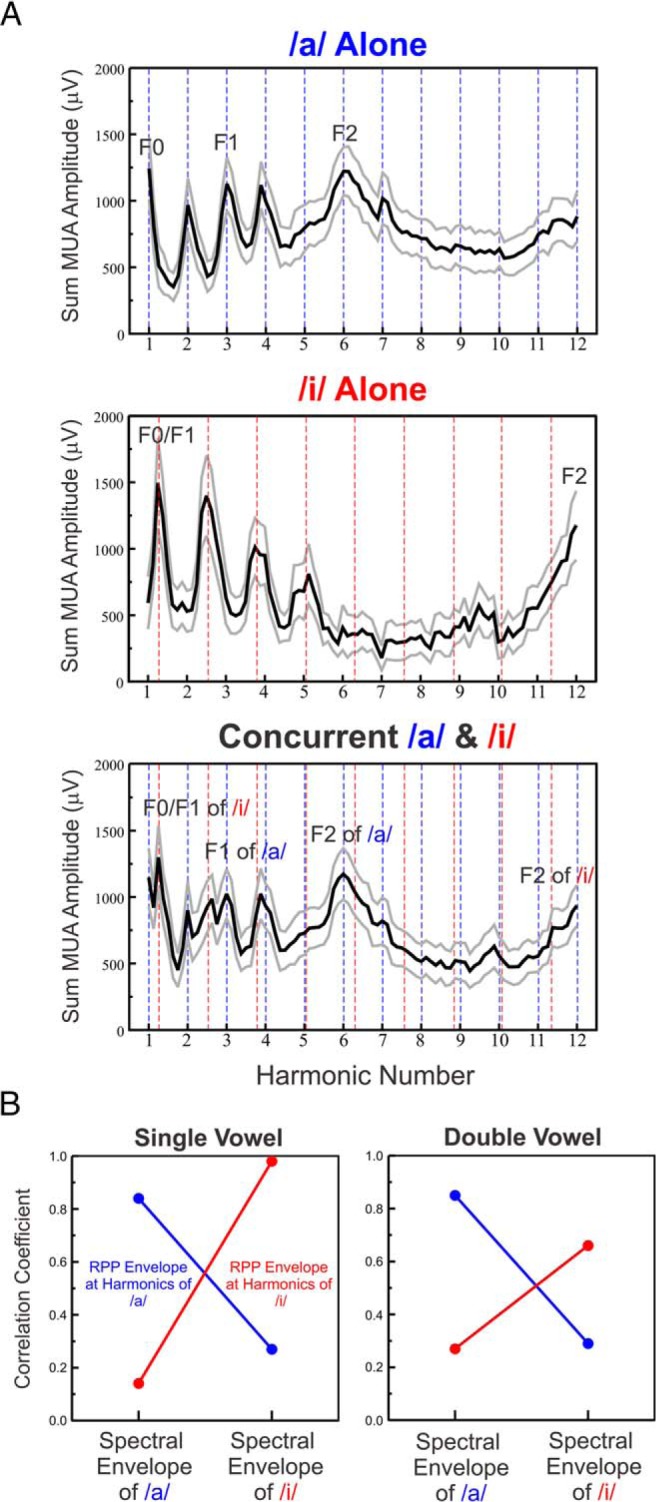
A. Rate-place profiles of responses to single and double concurrent vowels averaged across all recording sites. Mean ± SEM are represented by black and gray lines, respectively. B. Pearson correlation between envelopes of average rate-place profiles at harmonics of the vowels (left: single, right: double) and the spectral envelopes of the single vowel stimuli. Same conventions as in Figure 4.

The previous analyses indicated that rate-place representations at individual sites in A1 can resolve the lower harmonics of single and double concurrent vowels, and can be used to identify and discriminate them based on their formant structures. However, as large portions of tonotopically-organized A1 will be simultaneously activated by the vowels, an important question is whether these capacities persist when rate-place profiles are averaged across recording sites. Moreover, averaging rate-place profiles across recording sites provides an estimate of the mean population response, which may be more closely related to the auditory percept evoked by the stimuli than any of the individual site responses. As illustrated in Figure 6, averaged rate-place profiles elicited by single vowels show prominent peaks at the lower harmonics and formant frequencies of the vowels, which persist in the averaged rate-place profiles elicited by the double concurrent vowels (Fig. 6*A*). Moreover, as observed at individual sites, averaged rate-place profile envelopes at harmonics of each of the vowels are highly correlated with the spectral envelopes of the matching vowel stimuli and poorly correlated with the spectral envelopes of the non-matching vowel stimuli (Fig. 6*B*). Thus, the average pattern of neural activity across A1 is capable of representing both the lower harmonics and the formant structures of single and concurrent vowels with sufficient resolution to enable their segregation, identification, and discrimination.

**Figure 7.**
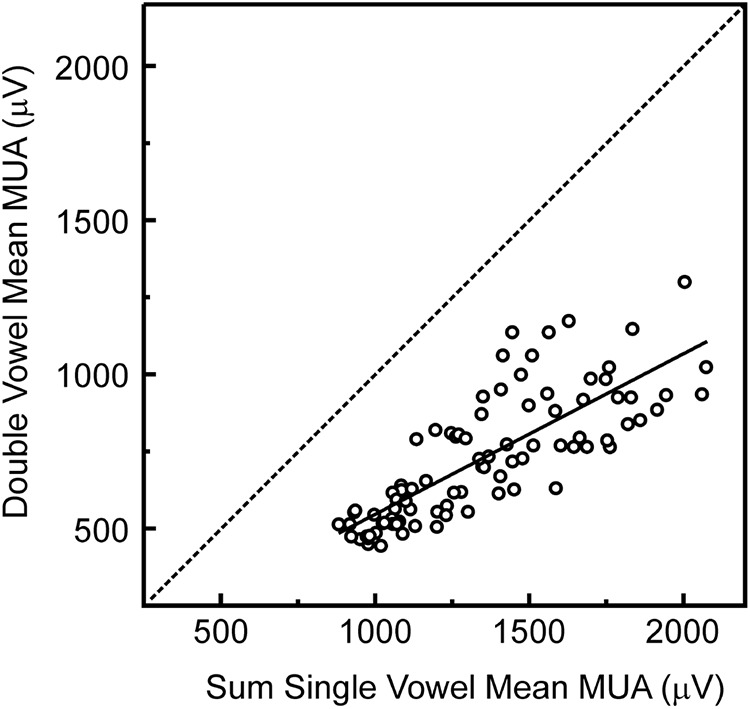
Nonlinearity of responses to double concurrent vowels. Sum of response amplitude at each harmonic value in the population average rate-place profile elicited by each of the single vowels is plotted against the response amplitude at the same harmonic values in the population average rate-place profile elicited by the double concurrent vowels (note that each rate-place profile is comprised of 89 amplitude values). A regression line fit to the data is superimposed. All values lie below the identity line, indicating that responses to double vowels are diminished compared with the sum of responses to the single vowels.

Finally, we sought to determine whether responses to double concurrent vowels in A1 reflect the linear sum of responses to the individual constituent vowels, or whether they exhibit nonlinearities, perhaps indicating the operation of inhibitory mechanisms, as suggested by previous studies of responses to complex sounds in A1 (David et al., 2009). Indeed, supportive of such mechanisms, we observed that responses to double concurrent vowels were of considerably lower amplitude than what would be expected based on a simple linear sum of responses to the individual vowels. This is demonstrated in Figure 7, where the sum of the response amplitudes at each harmonic value in the population average rate-place profile elicited by each of the single vowels is plotted against the response amplitude at the same harmonic values in the population average rate-place profile elicited by the double concurrent vowels. All values lie below the identity line, indicating that responses to double vowels are invariably diminished compared with the sum of responses to the single vowels.

## Discussion

The present study examined whether spectral information sufficient for extracting the F0s of two concurrently presented synthetic vowels, a prerequisite for their perceptual segregation, is available at the level of A1. In addition, we sought to determine whether neural population responses in A1 can discriminate and identify the single and double vowels based on their formant structures. F0s of the concurrent vowels differed by four semitones; an amount sufficient for them to be individually identified and reliably heard as two separate auditory objects with distinct pitches in human listeners (Assmann and Summerfield, 1990; Assmann and Paschall, 1998; Micheyl and Oxenham, 2010). To our knowledge, this is the first examination of the neural representation of double concurrent vowels in A1 of nonhuman primates.

We found that neural populations in A1 are able to resolve, via a rate-place code, lower harmonics (1–6) of each of the vowels comprising the double vowel stimuli. Importantly, evidence strongly suggests that resolvability of these lower harmonics is critical for pitch perception and for the ability to perceptually segregate complex sounds based on F0 differences in humans (Micheyl et al., 2010; Micheyl and Oxenham, 2010). Furthermore, we found that neural population responses in A1 can reliably discriminate and identify the single and concurrent vowels based on their spectral envelopes using a rate-place code. In general, A1 response amplitudes were proportional to the spectral energy of the vowel stimuli falling within the excitatory response areas (frequency response functions) of the neural populations, consistent with findings in previous studies examining complex sound encoding in A1 of both primate and non-primate species (Wang et al., 1995; Mesgarani et al., 2008; Qin et al., 2008). These findings thus suggest that the spectral fine-structure (lower harmonics) and formant structure (spectral envelopes) of single and double concurrent vowels are represented topographically as a rate-place code in A1 with sufficient resolution, reliability, and specificity to enable their perceptual segregation and identification. In principle, the pitches and identities of the vowels could be extracted from this spectral information via template-matching mechanisms (Goldstein, 1973; Assmann and Summerfield, 1990; de Cheveigné and Kawahara, 1999; Hillenbrand and Houde, 2003).

An important question is whether the responses to single and double concurrent vowels in A1 reflect neurophysiological properties that are unique to the cortex or properties that are inherited (perhaps in degraded form) from lower stations in the auditory pathway. In the auditory nerve, rate-place representations of single and concurrent vowels tend to saturate at higher sound levels (Sachs and Young, 1979; Palmer, 1990; Cedolin and Delgutte, 2010). Because of this limitation, it is generally thought that pitch encoding and vowel identification at these lower stations rely primarily on a temporal mechanism, ie, neuronal phase-locking to individual harmonics of the sounds and their F0s, which remains viable at higher sound levels (Palmer, 1990).

However, in the present study we found that rate-place representations of lower harmonics and formant structures of single and double vowels in A1 are highly robust at moderate-to-high sound levels (60 dB SPL per harmonic component). This suggests the operation of additional central mechanisms at, or prior to, the cortical level which enable reliable rate encoding of the harmonics and formant structures of single and concurrent vowels over a range of sound levels commonly encountered in real-world environments. Consistent with this suggestion, we observed that responses to double concurrent vowels were of considerably lower amplitude than what would be expected based on a simple linear sum of responses to the individual vowels (Fig. 7). This finding supports the involvement of inhibitory mechanisms, such as simultaneous (or “lateral”) suppression (Shamma and Symmes, 1985; Fishman and Steinschneider, 2006; Sadagopan and Wang, 2010; Fishman et al., 2012), which constrain the dynamic range of responses in A1, and which may contribute to previously reported nonlinearities observed in responses to complex sounds at the cortical level (Calhoun and Schreiner, 1998; Christianson et al., 2008; David et al., 2009; Gaucher et al., 2013; Mesgarani et al., 2014b). Indeed, it is noteworthy that the harmonics and formants of concurrent vowels can be reliably represented in A1 despite these nonlinearities.

Alternatively, the high fidelity of spectral fine-structure encoding in A1 may reflect a transformation of temporal representations of individual vowel components in peripheral structures into a rate code at higher levels of the auditory pathway (Buonomano and Merzenich, 1995; Wang et al., 2008). The auditory nerve and cochlear nucleus contain abundant temporal information from which, in principle, the pitch of vowels and other harmonic complex sounds may be derived (Palmer, 1990; Cariani and Delgutte, 1996; Keilson et al., 1997; Larsen et al., 2008; Cedolin and Delgutte, 2010; Sayles et al., 2015). Whereas peripheral auditory neurons can phase-lock to stimulus periodicities up to several thousand Hertz (Rose et al., 1967; Langner, 1992), a purely temporal code for pitch is not viable at the cortical level, where upper limits of phase-locking are too low (generally <200 Hz) to account for the full range of F0s characteristic of human pitch perception, as determined in human and nonhuman primate studies (Steinschneider et al., 1998; Brugge et al., 2009; Fishman et al., 2013, 2014; Steinschneider et al., 2013). Moreover, temporal codes for pitch might not be sufficiently resistant to effects of reverberation to enable concurrent vowel segregation based on F0 differences in real-world environments (Qin and Oxenham, 2005; Sayles et al., 2015). Hence, a temporal code for pitch would need to be either supplemented or replaced by a mechanism at the level of auditory cortex, which derives pitch from a rate-place representation of individual harmonics of complex sounds as described here for vowels and previously for harmonic complex tones with flat spectral envelopes (Fishman et al., 2013, 2014). The present findings suggest that neural populations in A1 can resolve the lower harmonics of single and double vowels with F0s as low as 129 Hz (775 Hz BF/6 harmonics; Fig. 3). Thus, the extent of spectral encoding of harmonics in A1 may indeed be adequate to cover the range of pitches examined in human psychoacoustic studies of concurrent vowel segregation (Assmann and Summerfield, 1990; Assmann and Paschall, 1998; Micheyl and Oxenham, 2010). Nonetheless, a temporal code for F0 may still be required to represent the much lower pitches that can be perceived by human listeners (Steinschneider et al., 1998; Plack et al., 2005).

Several limitations of the present study should be noted. First, monkeys were not engaged in tasks designed to evaluate whether they could segregate and identify the vowels. Thus, we cannot answer whether the A1 responses described here would parallel their perceptual performance in concurrent sound segregation and vowel identification tasks. However, several psychophysical studies report broad similarities between macaques and humans in vowel discrimination performance, suggesting that macaques may serve as a useful model of human vowel discrimination, at least in the case of single isolated vowels (Sinnott, 1989; Sommers et al., 1992; Sinnott et al., 1997). Second, because only two vowels and a single F0 difference were tested, it remains unclear whether the present results are representative of responses to an arbitrary set of single and concurrent vowels with different F0 separations. However, our previous study using harmonic complexes with flat spectral envelopes (Fishman et al., 2014) suggests that harmonic resolvability is comparatively poor when F0 difference is one semitone and improves with increasing F0 difference up to four semitones, paralleling psychoacoustic data in humans (Assmann and Paschall, 1998). Thus, it is reasonable to expect similarly reduced harmonic resolvability for concurrent vowels with smaller F0 differences. As the two vowels /a/ and /i/ lie close to the extremes of the spectral envelope continuum for English vowels, the present findings may be regarded as providing a conservative upper bound on the extent to which single and double vowels may be discriminated and identified based on neural population responses in A1. Third, because the present results are based on the responses of small neuronal populations, rather than individual neurons, they may underestimate the capacity of A1 to resolve the harmonics and formant structures of single and double vowels. Nonetheless, the present demonstration that stimulus features relevant for pitch perception and vowel identification can be reliably represented by population responses in A1 is important, given that synchronized activity of neural ensembles in auditory cortex may convey pitch-related information more robustly than single-unit responses (Bizley et al., 2010) and may be more likely to drive neurons in downstream cortical areas putatively involved in pitch extraction and vowel identification to firing threshold (Eggermont, 1994; deCharms and Merzenich, 1996; Tiesinga et al., 2008; Wang et al., 2010). A final limitation is that the F0s of the vowel stimuli used in the present study were varied at each site such that the BF occupied different values of harmonic number with respect to the F0 of the sounds. The rate-place profiles generated from responses elicited using this stimulus design provide only an indirect measure of neural resolvability in A1. However, by invoking the principle of “scaling invariance” (Larsen et al., 2008), whereby the ratio of frequency tuning bandwidth to BF at each site is constant across sites, the present data can be used to infer the response to the single and double vowel stimuli across sites in A1. Previous findings based on responses to harmonic complex tones with flat spectral envelopes indicate that scaling invariance holds to a good approximation in A1, with a slight departure from strict scaling invariance being primarily due to sites with higher BFs having narrower relative tuning bandwidths than sites with lower BFs (Fishman and Steinschneider, 2009; Fishman et al., 2013). This narrower tuning may partly explain why higher BF sites are better able to resolve harmonics of the vowels than lower BF sites (Fig. 3). Consequently, the extent of neural resolvability of harmonics at higher (lower) BF sites would be slightly underestimated (overestimated) if inferred based on rate-place profiles obtained at lower (higher) BF sites. Nonetheless, the scaling invariance assumption is generally valid when applied over a local region of BFs covered by the range of harmonics comprising the vowel stimuli used in the present study. Consequently, measures of neural resolvability obtained at individual sites will be largely representative of the resolvability of double HCT harmonics across sites in A1.

In conclusion, the present findings indicate that A1 contains sufficient spectral information to promote the perceptual segregation of concurrent vowels based on differences in F0 and their subsequent identification based on their formant structure. In principle, the pitches and identities of the vowels could be extracted via template-matching mechanisms (Goldstein, 1973; Assmann and Summerfield, 1990; de Cheveigné and Kawahara, 1999; Hillenbrand and Houde, 2003) operating on the spectral fine-structure and envelope information, respectively, in higher-order brain regions that receive input from A1 (Bendor and Wang, 2005; Obleser et al., 2006; Uppenkamp et al., 2006; Mesgarani et al., 2014a). Exactly how this spectral information is used by downstream cortical areas for pitch extraction and vowel identification, and how top-down mechanisms subsequently modulate responses to attended versus unattended speech streams in multi-talker environments (Ding and Simon, 2012; Mesgarani and Chang, 2012; Zion-Golumbic et al., 2013; Kong et al., 2015) remain important questions to be explored in future studies.

## Synthesis

The decision was a result of the Reviewing Editor Tatyana Sharpee and the peer reviewers coming together and discussing their recommendations until a consensus was reached. A fact-based synthesis statement explaining their decision and outlining what is needed to prepare a revision is listed below.

Responses to previous reviews were adequately addressed.
